# Hallucinations and disturbed behaviour in the critically ill: incidence, patient characteristics, associations, trajectory, and outcomes

**DOI:** 10.1186/s13054-025-05290-1

**Published:** 2025-01-31

**Authors:** Thomas Niccol, Marcus Young, Natasha E. Holmes, Kartik Kishore, Sobia Amjad, Michele Gaca, Rinaldo Bellomo, Ary Serpa Neto

**Affiliations:** 1 Australian and New Zealand Intensive Care Research Centre, Monash University, Melbourne, Australia; 2 Department of Critical Care, Melbourne University, Melbourne, Australia; 3 Data Analytics Research and Evaluation Centre, Austin Hospital, Melbourne, Australia; 4 Department of Intensive Care, Austin Hospital, 145 Studley Rd, Heidelberg, Melbourne, VIC Australia; 5 Department of Infectious Diseases, University of Melbourne, Peter Doherty Institute for Infection and Immunity, Melbourne, VIC 3000 Australia; 6 Department of Intensive Care, Royal Melbourne Hospital, Melbourne, Australia; 7 Department of Critical Care Medicine, Hospital Israelita Albert Einstein, São Paulo, Brazil; 8 Department of Surgery, School of Medicine, The University of Melbourne, Parkville, Melbourne, VIC Australia; 9 School of Computing and Information Systems, The University of Melbourne, Parkville, Melbourne, VIC Australia

**Keywords:** Intensive care, Hallucinations, Delirium, Critical illness, Mortality, Antipsychotic drugs

## Abstract

**Purpose:**

To use natural language processing (NLP) to study the incidence, characteristics, trajectory, associations, and outcomes of hallucinations and disturbed behaviour in intensive care unit (ICU) patients.

**Methods:**

We used NLP to scan clinical progress notes of a large cohort of ICU patients to detect words indicating that a patient had experienced hallucinations. We also used NLP to detected disturbed behaviour during ICU stay. Moreover, we studied the use of antipsychotic medications in a nested cohort. Finally, we obtained the demographics, trajectory, associations, and outcome of these patients.

**Results:**

We conducted a non-interventional, observational study of 7525 patients. We found that 625 (8.31%) had experienced hallucinations. Among these, 623 (99.7%) also had NLP-diagnosed behavioural disturbance (NLP-Dx-BD). In contrast, in patients without hallucinations, only 3274 (47.4%) were NLP-Dx-BD positive. Among the 2904 nested cohort patients with electronic medications data, 252 (8.7%) experienced hallucinations. Of these, 60 (23.8%) received medications compared with 147 (5.5%) (*p* < 0.001) patients without hallucinations. There was no difference on outcomes in patients with or without hallucination.

**Conclusions:**

Hallucinations affect one in 12 ICU patients and are strongly associated with disturbed behaviour, and the use of antipsychotic medications. Hallucinations may represent another phenotype of critical illness associated neurocognitive dysfunction and require a dedicated research program.

**Supplementary Information:**

The online version contains supplementary material available at 10.1186/s13054-025-05290-1.

## Introduction

The fifth edition of the Diagnostic and Statistical Manual of Mental Disorders defines hallucinations as “a perception-like experience with the clarity and impact of a true perception but without the external stimulation of the relevant sensory organ” [1]. Such hallucinations may occur with some frequency in critically ill patients and may be distressing and frightening. They may also induce or be associated with disturbed behaviour and fear, and trigger the use of sedative or antipsychotic medications [2]. Finally, hallucinations may represent the early warning signs of delirium and thus be useful triggers for prophylactic interventions. However, although these considerations reflect clinical experience, evidence on hallucinations in ICU patients is limited and hampered by methodological problems.

All studies of hallucinations in ICU have, so far, relied on post-discharge interviews to establish a patient’s recall of episodes of hallucinations during their ICU stay [3–9]. Such post-hoc analyses are subject to autosuggestion, memory loss, flawed recollections, leading question bias, and immortal time bias. Within the framework created by such limitations, the rate of hallucinations in critically ill patients who have also been screened as positive for delirium has been reported to be as high as 65% [10]. However, other post-hoc studies have been contradictory and reported no such association in mechanically ventilated patients [11]. This has generated scepticism and confusion in this field of research. In this regard, a possible approach to overcome such methodological challenges is one based on the use of Natural Language Processing (NLP).

NLP is a powerful screening tool and has been previously used to identify critically ill patients with disturbed behaviour [12–15]. Patients screened positive for disturbed behaviour using NLP have been described as having natural language processing diagnosed behavioural disturbance (NLP-Dx-BD) [16]. Such screening has been demonstrated to align with alternative screening techniques such as the confusion assessment method for the intensive care unit (CAM-ICU) [12]. Thus, NLP enables the analysis of contemporaneous observations of care givers as recorded in the clinical progress notes, an option not available through alternate screening techniques or post-hoc interviews. As such, NLP can be applied to the study of hallucinations in the same way that it has been applied to the study of delirium.

Accordingly, we hypothesised that words and phrases indicative of a patient experiencing hallucinations may be contemporaneously recorded by care givers in the clinical progress notes. We further hypothesised that natural language processing (NLP) could search these notes for such words and provide information on the incidence of hallucinations and on the characteristics, trajectory, associations, and outcomes of patients experiencing them.

## Methods

### Study design

We conducted a non-interventional, observational study of critically ill patients admitted to three university affiliated medical-surgical intensive care units. We included only adult patients (≥ 18 years old) with a minimum length of stay of more than 4 h and at least one clinical progress note. No other exclusion criteria were applied. The study was approved by the Austin Hospital Human Research Ethics Committee (LNR/19/Austin/38), which waived the requirement for informed consent.

### Data collection and manipulation

We obtained the electronic clinical progress notes recorded by doctors, nurses, and allied health professionals. Further, we obtained the medication records from the hospital’s electronic medication management system for a nested cohort of patients admitted to our intensive care units from March 1, 2019, to December 31, 2020. We obtained baseline information and outcome data from the Australian and New Zealand Intensive Care Society Adult ICU Patient Database run by the Centre for Outcome and Resource Evaluation [17].

Using a previously described method [16] each note was converted into sentence vectors and each sentence vector into a set of tokens (Natural Language Toolkit; NLTK 3.5) [18]. We then scanned each token set to identify key words that may indicate that a patient had experienced hallucinations or disturbed behaviour during the period for which the note applied (Appendix eTable 1). We further checked each identified key word for negation or resolution (for example “not agitated”) (Appendix eTable 1). The majority of the key words used in this study were derived from a previously published survey of words used by doctors, nurses and allied health professionals to describe disturbed behaviour [19]. However, for the present study, the word list was augmented by words indicative of the patient experiencing hallucinations Morphemes and alternate spellings were managed by scanning for variants of the same word and, in order to deal with typing errors (e.g., halucinations or halucinate) (Appendix eTable 1), by using stemming where the roots of words rather than the roots and associated suffixes were scanned. Moreover, using NLP, we analysed the bedside notes for documentary evidence of the presence of schizophrenia and/or bipolar disorder [20].

### Exposure

The primary exposure of this study for an individual patient was the presence in a progress note of a word or words indicating that they had experienced hallucinations or disturbed behaviour during any 24 h period through their stay in ICU. All patients within the study received care designed to reduce the risk of developing delirium and neurological disturbances. This included family visits, dimmed lights at night, minimal interaction to facilitate night-time sleep, and the use of visual and auditory aids as required.

### Outcomes

The primary outcome of this study was the incidence of hallucinations. The secondary outcomes included the incidence of NL-Dx-BD in patients with hallucinations, the use of medications in patients with hallucinations, the duration of reported hallucinations, and the duration of ICU and hospital stay as well as mortality rate at hospital discharge and day 28.

### Statistical analysis

All continuous data are reported as median with interquartile range (IQR) and categorical data as number and percentage. In the primary descriptive analysis, data from all patients fulfilling inclusion criteria were reported according to the presence (or absence) of hallucination. No missing data for any of the outcomes were present in the dataset; therefore, all analyses were complete case analyses. Baseline and clinical characteristics of the patients were compared among the groups using Fisher exact tests and Wilcoxon rank sum tests.

A multivariable time-dependent Cox proportional hazard model was used to assess the impact of hallucination on outcomes while accounting for immortal bias. In addition, to account for competing events when assessing duration of ventilation, and ICU and length of stay, failure to be extubated or discharged and death were both censored at the longest (‘worst’) follow-up. All models were adjusted by age, type of admission and by the Australian and New Zealand Risk of Death (ANZROD) after log transformation [21]. As previously shown, ANZROD is a powerful predictor and explains most of the mortality in ICUs in Australia and New Zealand. In addition, ANZROD is superior to the Acute Physiology and Chronic Health Evaluation (APACHE) III scores in predicting mortality in Australia and New Zealand, with an area under the receiver operating characteristic curve (AUROC) of 0.902 [22]. Time to hallucination is reported in cumulative incidence plots considering ICU mortality and discharged alive as competing events. We also investigated baseline characteristics potentially associated with development of hallucination using a multivariable logistic regression model including baseline characteristics that were found to be associated with hallucination in an univariable assessment. Finally, a sensitivity analysis was performed excluding patients who received mechanical ventilation during ICU stay to reduce the risk of including patients under deep sedation or unable to communicate.

All analyses were conducted in R v.4.0.2 (R Foundation for Statistical Computing, Vienna, Austria) and *P* < 0.05 was considered statistically significant.

## Results

### Incidence

We studied 7525 critically ill patients admitted to our intensive care units between 11 July 2016 and 22 May 2022. The baseline characteristics of patients with and without hallucinations within this cohort are presented in Table 1. In this large cohort with very low data missingness (Appendix eTable 2), we identified that 625 patients had experienced hallucinations in ICU. In 281 patients with hallucinations (45.0%), more than one episode was recorded during follow-up and the median hours until resolution of the first episode of hallucination was 15 (12–18) hours (Appendix eTable 3).

**Table 1:** Baseline characteristics of the included patients

	Overall (*n* = 7525)	Hallucination (*n* = 625)	No hallucination (*n* = 6900)	*p* value
Age, years	63.7 (51.1–74.1)	61.4 (47.8–72.2)	64.0 (51.5–74.2)	< 0.001
Male gender—no. (%)	4619/7519 (61.4)	386 (61.8)	4233/6894 (61.4)	0.928
Body mass index, kg/m^2^	27.7 (24.0–32.2)	27.0 (23.9–31.8)	27.8 (24.0–32.3)	0.438
APACHE III	48.0 (35.0–64.0)	55.0 (39.0–73.0)	47.0 (34.2–63.0)	< 0.001
ANZROD	2.4 (0.7–10.0)	4.1 (1.3–16.1)	2.3 (0.7–9.4)	< 0.001
Type of admission—no. (%)				0.155
Medical	3854/7522 (51.2)	337/624 (54.0)	3517/6898 (51.0)	
Surgical	3668/7522 (48.8)	287/624 (46.0)	3381/6898 (49.0)	
Admitted from mental health facilities—no. (%)	27 (0.4)	4 (0.6)	23 (0.3)	0.278
Planned admission—no. (%)	2255 (30.0)	152 (24.3)	2103 (30.5)	0.001
MET call admission—no. (%)	1300/7524 (17.3)	116/624 (18.6)	1184 (17.2)	0.376
Cardiac arrest—no. (%)	198/7514 (2.6)	14/624 (2.2)	184/6890 (2.7)	0.603
Acute renal failure—no. (%)	216/7471 (2.9)	47/621 (7.6)	169/6850 (2.5)	< 0.001
Alcohol withdrawal—no. (%)	4/7325 (0.1)	1/605 (0.2)	3/6720 (0.0)	0.292
Admission diagnosis—no. (%)				< 0.001
Cardiovascular	2492/7522 (33.1)	142/624 (22.8)	2350/6898 (34.1)	
Gastrointestinal	1280/7522 (17.0)	176/624 (28.2)	1104/6898 (16.0)	
Gynaecological	14/7522 (0.2)	1/624 (0.2)	13/6898 (0.2)	
Haematological	68/7522 (0.9)	8/624 (1.3)	60/6898 (0.9)	
Metabolic	459/7522 (6.1)	40/624 (6.4)	419/6898 (6.1)	
Musculoskeletal/Skin	159/7522 (2.2)	17/624 (2.8)	142/6898 (2.1)	
Neurological	642/7522 (8.5)	59/624 (9.5)	583/6898 (8.5)	
Renal/Genitourinary	361/7522 (4.8)	19/624 (3.0)	342/6898 (5.0)	
Respiratory	1078/7522 (14.3)	73/624 (11.7)	1005/6898 (14.6)	
Sepsis	708/7522 (9.4)	54/624 (8.7)	654/6898 (9.5)	
Trauma	261/7522 (3.5)	35/624 (5.6)	226/6898 (3.3)	
ICU source of admission—no. (%)				< 0.001
Emergency department	1779 (23.6)	129 (20.6)	1650 (23.9)	
Operating room	3645 (48.4)	287 (45.9)	3358 (48.7)	
Ward	1215 (16.1)	106 (17.0)	1109 (16.1)	
ICU other hospital	149 (2.0)	28 (4.5)	121 (1.8)	
Other hospital	724 (9.6)	74 (11.8)	650 (9.4)	
ICU same hospital	6 (0.1)	1 (0.2)	5 (0.1)	
Other	7 (0.1)	0 (0.0)	7 (0.1)	
*Co-existing disorders—no. (%)*				
Diabetes	1059/1344 (78.8)	87/114 (76.3)	972/1230 (79.0)	0.475
Chronic lung disease	737 (9.8)	58 (9.3)	679 (9.8)	0.725
Chronic cardiovascular disease	339 (4.5)	34 (5.4)	305 (4.4)	0.228
Cirrhosis	560 (7.4)	84 (13.4)	476 (6.9)	< 0.001
Chronic kidney disease	679 (9.0)	69 (11.0)	610 (8.8)	0.068
Chronic immune disease	192 (2.6)	20 (3.2)	172 (2.5)	0.288
Immunosuppression	585 (7.8)	54 (8.6)	531 (7.7)	0.391
Hepatic failure	104 (1.4)	15 (2.4)	89 (1.3)	0.031
Lymphoma	83 (1.1)	6 (1.0)	77 (1.1)	1.000
Metastatic cancer	330 (4.4)	28 (4.5)	302 (4.4)	0.919
Leukemia	153 (2.0)	14 (2.2)	139 (2.0)	0.657
Bipolar disorder	90 (1.2)	19 (3.0)	71 (1.0)	< 0.001
Schizophrenia	117 (1.6)	30 (4.8)	87 (1.3)	< 0.001
*Organ support—no. (%)*				
ECMO	17/5193 (0.3)	4/467 (0.9)	13/4726 (0.3)	0.060
Vasopressor or inotropes	2697/5195 (51.9)	305/467 (65.3)	2392/4728 (50.6)	< 0.001
Invasive ventilation	3906/6334 (61.7)	415/556 (74.6)	3491/5778 (60.4)	< 0.001
Non-invasive ventilation	343/5224 (6.6)	33/469 (7.0)	310/4755 (6.5)	0.626
Renal replacement therapy	489/5331 (9.2)	107/486 (22.0)	382/4845 (7.9)	< 0.001
*Laboratory tests*				
pH	7.38 (7.32–7.43)	7.36 (7.30–7.42)	7.38 (7.32–7.43)	< 0.001
PaO_2_/FiO_2_	305 (212–400)	280 (190–386)	309 (215–403)	< 0.001
PaCO_2_, mmHg	40 (35–45)	41 (36–45)	40 (35–45)	0.464
Lactate, mmol/L	2.1 (1.5–3.2)	2.4 (1.7–4.2)	2.0 (1.4–3.1)	< 0.001
Highest creatinine, µmol/L	91 (69–138)	102 (72–165)	91 (69–135)	< 0.001
Lowest platelet, × 10^9^/L	176 (125–239)	160 (99–233)	177 (126–239)	< 0.001
*Vital signs*				
Lowest MAP, mmHg	65 (59–72)	65 (58–71)	65 (59–73)	0.124
Highest RR, breaths/min	20 (15–25)	20 (16–25)	20 (15–25)	0.190
Highest temperature, ºC	37.2 (36.7–37.6)	37.3 (36.8–37.8)	37.2 (36.6–37.5)	< 0.001
Urine output, mL	1509 (1085–2140)	1435 (921–2025)	1517 (1095–2150)	0.002

Data are median (IQR) or N (%)

*APACHE* is Acute Physiology and Chronic Health Evaluation, *MET* is medical emergency team, *ICU* is intensive care unit, *ECMO* is extracorporeal membrane oxygenation, *MAP* is mean arterial pressure, *RR* is respiratory rate

Using NLP and analysing a median of 11 notes per patients (Appendix eTable 3), we found that the 625 (8.31%) patients with hallucinations had specific characteristics. They were younger, more acutely ill, more likely to be an unplanned admission to ICU, more likely to be admitted with gastrointestinal illness, more likely to have pre-existing cirrhosis and/or hepatic failure. Further, patients in this group were more likely to have acute kidney injury (AKI), and require vasopressor or inotropic support, invasive ventilation, and renal replacement therapy. The number of patients admitted from mental health facilities was similar between the groups. The number of patients with bipolar disorder or schizophrenia was higher in the group of patients who experienced hallucinations. Among 362 episodes of hallucinations where more information was available, 250 (69.1%) were visual, 51 (14.1%) were auditory and 61 (16.8%) were both auditory and visual.

### Trajectory

**Fig. 1:**
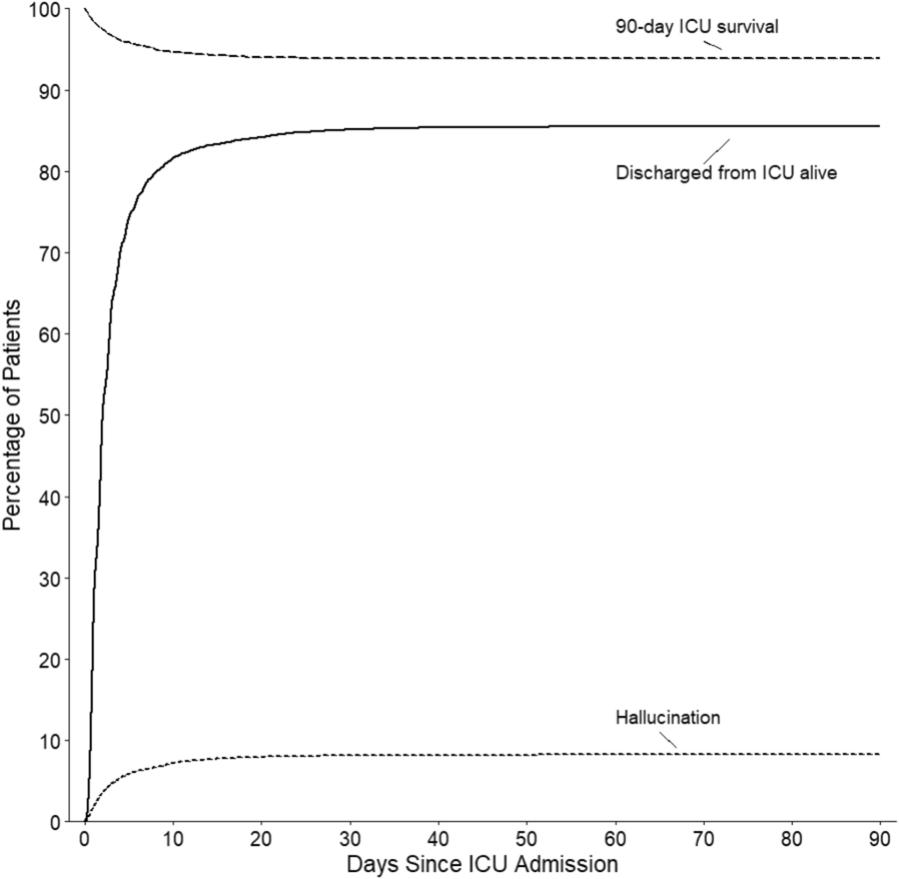
Cumulative incidence plot of hallucination. Cumulative incidence plot of the first episode of hallucination considering ICU mortality and discharged alive as competing events

**Fig. 2:**
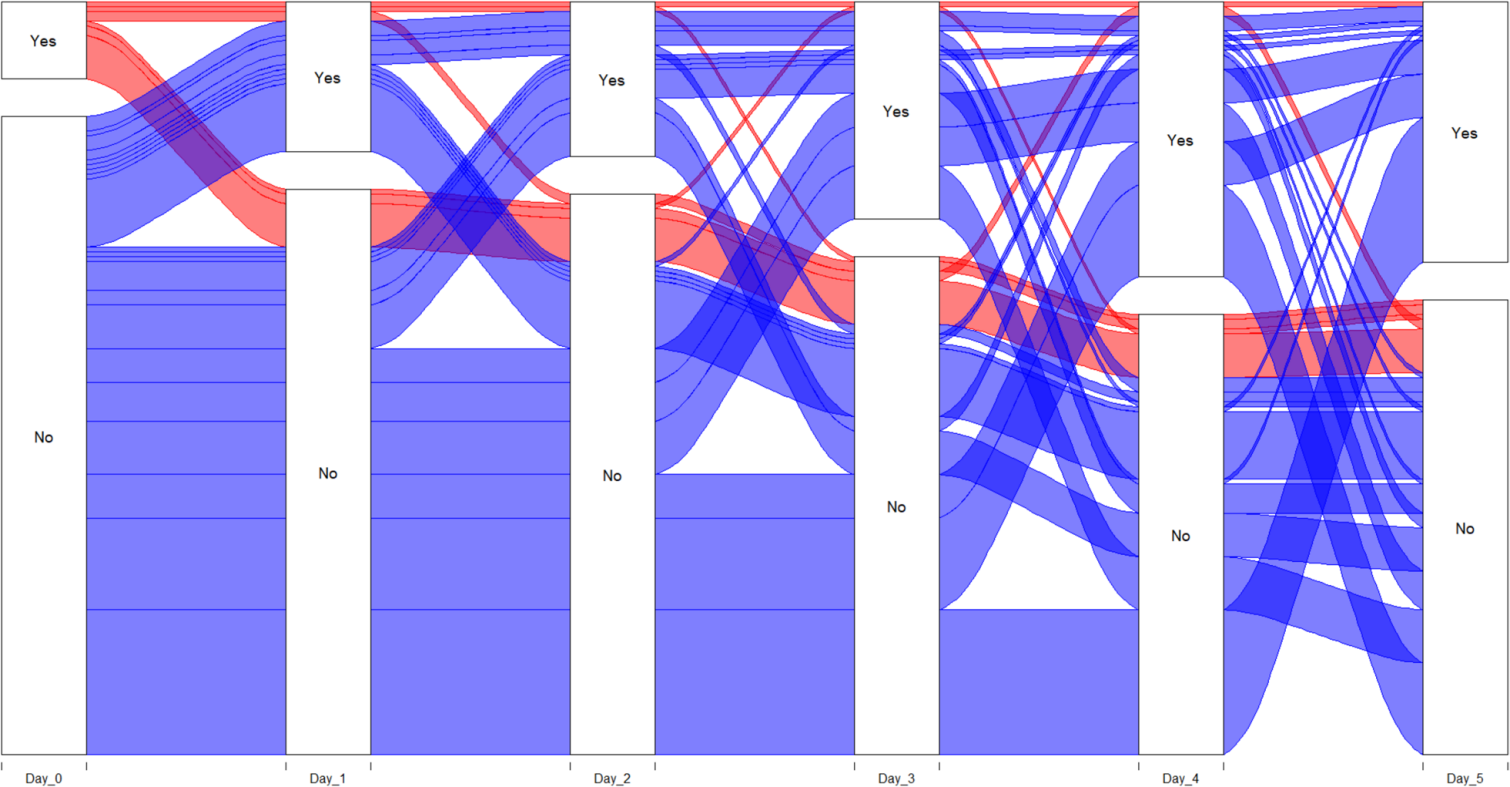
Trajectory of hallucination during the first 5 days. Alluvial plot demonstrating the trajectory of hallucinations in patients who experience them in ICU. Blue are patients without hallucination on day 0 and red are patients with hallucination on day 0

Hallucinations were most common on the day after ICU admission with the majority occurring within the first three days (Fig. 1). The alluvial plot shown in Fig. 2 describes the trajectory of all patients who developed at least one episode of hallucinations ICU. It shows that approximately one third of patients with hallucinations on day one continued to experience hallucinations on day two and that approximately two thirds of such patients achieved resolution by day five. On the other hand, approximately one of every six patients who were free of hallucinations on day one or day two went on to develop hallucinations on the following day, with a similar rate of resolution over time.

### Association with disturbed behaviour

As shown in Table 2, among patients with hallucinations, 99.7% screened positive NLP-Dx-BD and 55.4% screened positive for delirium. These rates were markedly higher than for patients without hallucinations (*P* < 0.001). Among patients with hallucinations, NLP-Dx-BD occurred on the same day of a hallucination in 613 (98.1%) and on the following day in 227 (36.2%) (Table 2). In a nested subgroup of 2904 patients admitted between March 1, 2019, and December 31, 2020, with the ability to obtain medications data from the Electronic Medical Record (EMR), we found similar baseline characteristics (Appendix eTable 4) to the larger study cohort. Also, like in the larger cohort, in this nested cohort, 252 (8.8%) patients experienced hallucinations and presented similar outcomes as in the main comparison (Appendix eTable 5). Of these 252 patients, 60 (23.8%) received antipsychotic medications while in ICU (Appendix eTable 6). In contrast, only 147 (5.5%) patients without hallucinations received such antipsychotic medications (*p* < 0.001). Among patients with hallucinations who received atypical antipsychotic medications, 27 (45%) started such treatment after the first episode of hallucinations and among the 30 patients who received haloperidol, nine (30%) received such treatment after the first episode of hallucinations (Appendix eTable 6). The use of ketamine was much higher in patients who developed hallucination (25.4% versus 6.9%; *p* < 0.001) and almost all patients (96.9%) received ketamine before the first episode of hallucination (Appendix eTable 6). The use of dexmedetomidine in this group is reported in Appendix eTable 6.

**Table 2:** Overlap between hallucinations and NLP-Dx-BD and delirium and timing of hallucinations in relation to NLP-Dx-BD

	Hallucination (*n* = 625)	No hallucination (*n* = 6900)
*NLP-Dx-BD*		
No	2/625 (0.3)	3626/6900 (52.6)
Yes	623/625 (99.7)	3274/6900 (47.4)
*NLP-Dx-BD*		
Same day of hallucination*	613/625 (98.1)	–
Next day of hallucination*	227/590 (38.5)	–
Same or next day of hallucination*	579/590 (98.1)	–
*Delirium*		
No	279/625 (44.6)	6099/6900 (88.4)
Yes	346/625 (55.4)	801/6900 (11.6)
*Delirium*		
Same day of hallucination*	92/625 (14.7)	–
Next day of hallucination*	69/590 (11.7)	–
Same or next day of hallucination*	137/590 (23.2)	–

*Considering the first day of hallucination

### Outcomes

As shown in Table 3, no statistically significant difference on outcomes was found in patients with hallucinations.

**Table 3:** Clinical outcomes of included patients

	Overall (*n* = 7525)	Hallucination (*n* = 625)	No hallucination (*n* = 6900)	Unadjusted models		Adjusted models^a^	
				Effect estimate (95% CI)	*p* value	Effect estimate (95% CI)	*p* value
Duration of ventilation, days*	1.0 (0.4–3.6)	3.8 (1.3–9.7)	0.9 (0.4–2.9)	HR, 1.17 (0.16 to 8.34)	0.876	HR, 1.44 (0.20–10.34)	0.715
ICU length of stay, days	1.9 (1.0–3.8)	5.0 (2.8–10.5)	1.8 (0.9–3.4)	HR, 1.16 (0.92 to 1.47)	0.218	HR, 1.03 (0.82–1.31)	0.775
Hospital length of stay, days	10.0 (5.7–18.9)	19.9 (10.4–33.7)	9.3 (5.4–17.3)	HR, 0.92 (0.72 to 1.18)	0.509	HR, 0.95 (0.74–1.21)	0.671
Hospital mortality—no. (%)	686 (9.1)	43 (6.9)	643 (9.3)	HR, 0.69 (0.29 to 1.66)	0.409	HR, 0.84 (0.35–2.04)	0.706
28-day mortality—no. (%)	601 (8.0)	28 (4.5)	573 (8.3)	HR, 0.78 (0.29 to 2.09)	0.622	HR, 0.82 (0.30–2.18)	0.686

Data are median (quartile 25^th^—quartile 75th) or N (%)

*ICU* is intensive care unit, *HR* is hazard ratio

*Duration of ventilation reported only in patients who received ventilation

^a^All models adjusted for age, type of admission and ANZROD (after logarithmic transformation)

### Characteristics associated with the development of hallucination

Baseline characteristics independently associated with the development of hallucination during ICU stay are reported in Appendix eTable 7. Presence of acute renal failure, liver cirrhosis, use of mechanical ventilation and renal replacement therapy, a high lactate, a high temperature and an admission due to gastrointestinal, musculoskeletal/skin, metabolic, neurological or trauma reason were associated with an increased risk of hallucination during ICU stay.

### Sensitivity analysis

Results of the sensitivity analysis are reported in Appendix eTables 8 and 9 and did not materially change the findings compared to the main analyses. In addition, Appendix eTable 10, we provide typical examples of notes used to identify the occurrence of hallucinations, which demonstrate that under almost all circumstances, their presence reflects information provided by the patient to the nurse, rather than the interpretation of patient observation.

## Discussion

### Key findings

We used Natural Language Processing (NLP) to screen bedside clinical progress notes of critically ill patients to identify patients who experienced hallucinations. Moreover, we used NLP to screen patients for NLP diagnosed disturbed behaviour (NLP-Dx-BD). We found that one in twelve patients experienced hallucinations and that such patients had specific characteristics consistent with more underlying liver disease and greater illness severity. Hallucinations occurred early during ICU admission and were strongly associated with the development of NLP-Dx-BD. Thus, among these patients, almost all screened positive for NLP-Dx-BD. The timing of such disturbed behaviour was either contemporaneous with or closely after the onset of hallucinations. Moreover, a quarter of patients with hallucinations received antipsychotic medications while in ICU. Finally, patients who experienced hallucinations had significantly longer ICU and hospital stays but were more likely to survive to hospital discharge.

### Relationship to previous studies

There are very few previous studies reporting on hallucinations in critically ill patients. In a study involving 289 ICU patients, Rundshagen et al. [3] conducted interviews 48–72 h after ICU discharge. Among such patients, 6.6% reported hallucinations while in ICU. The incidence reported in our study is broadly aligned with such data. These investigators did not provide additional data on patients with hallucinations. However, in a retrospective study of a group of 602 general ward patients with delirium, Tachibana et al. [22] reported that 25.9% had experienced hallucinations, with visual hallucinations being most common (92.3%). A background of alcohol abuse and benzodiazepine withdrawal were found to be significantly associated with hallucinations in this setting. Our observation of a greater incidence of liver disease and cirrhosis in our cohort is aligned with such findings. Similarly, the overlap between delirium and hallucinations seen in this study is also consistent with our observations. Van de Leur et al. [4] interviewed a cohort of 132 mechanically ventilated patients and found that 24.2% reported hallucinations. However, when their factually verifiable recollection performance was tested, correct recall was below 70% for all factual items, highlighting the problems associated with the retrospective diagnosis of hallucinations based on patient recall. Finally, Smit et al. reported that 80% of ICU patients in a trial of haloperidol had either hallucinations or delusion. However, these two conditions were not separated [2]. Thus, ours is the first study to analyse the contemporaneous observation of patient behaviour and statements recorded by caregivers in bedside clinical notes and to use such notes to identify patients with hallucinations. Finally, our study is one order of magnitude larger than previous investigations.

### Implications of study findings

Our study implies that NLP can be used to identify critically ill patients with hallucinations. It further implies that NLP can be used to conduct large scale epidemiological studies of studies of hallucinations by analysing vast repositories of electronic bedside progress notes. Such methodology also demonstrates that most patients with hallucinations also demonstrate other disturbances in neurocognitive function and behaviour. This finding suggests that hallucinations may be another manifestation or phenotype of the complex neurocognitive dysfunction of critical illness. Finally, our findings demonstrate an association between hallucinations and treatment with antipsychotic medications and the occurrence of NLP-Dx-BD after the onset of hallucinations in a significant proportion of such patients. This observation suggests the need to investigate whether effective treatment can be deployed to prevent or attenuate such hallucinations and mitigate the transition to behavioural disturbance.

### Strengths and limitations

Our study has several strengths. We used a tool (NLP-based diagnosis), which we have previously applied and validated of the diagnosis of behavioural disturbances, likely delirium and delirium subtypes, thus providing face validity for the use of such AI technology in ICU [12, 15, 16]. Accordingly, it is the first study to use NLP to identify episodes of hallucinations in critically ill patients. Further, it is the first study to investigate hallucinations in a large cohort of critically ill patients using contemporaneously documented caregiver observations rather than post-hoc interviews, with their attendant inaccuracies and biases. Moreover, to the best of our knowledge, it provides the first data detailing the association between episodes of hallucinations and disturbed behaviour in critically ill patients. Finally, it provides insight into the association between treatment with antipsychotic medications, ketamine, hallucinations, and disturbed behaviour and, for the first time, information on the outcome of such patients. Such information can be used to design interventional study of therapy and prevention in patients at risk of or with hallucinations.

We acknowledge several limitations. First, our study was undertaken in the intensive care units of a university affiliated tertiary hospital in a resource-rich country. Therefore, our findings may not apply to other intensive care units in low or middle-income countries. Further, other intensive care departments may use strategies for managing hallucinations and disturbed behaviour that result in different rates and outcomes. In our study, patients were not assessed independently for the presence of hallucinations. However, the terms we used to identify episodes of hallucinations were highly specific and more likely to underestimate its incidence. In this regard, the diagnosis of hallucinations still ultimately relies on patients reporting their occurrence to the nursing, allied health, or medical staff. It is also possible that nurses would infer the presence of hallucinations where they do not actually exist. However, a detailed analysis of notes, as reported in the supplementary appendix, confirms that, in almost all cases, such hallucinations were actually reported by the patients themselves. Thus, in the absence of other diagnostic tools, it is likely that the incidence of hallucinations will remain underestimated. The associated with ketamine may be spurious. Knowledge that patients were receiving ketamine might have triggered more detailed questioning and searching for hallucinations. Thus, this association requires more investigation. The reporting of hallucinations implies the ability of the patient to communicate and creates a type of selection bias. Such ability to communicate identifies patients who although initially more acutely ill are on a trajectory to improvement and may explain both their longer ICU stay. Also, we do not have specific information on ICU-related variables that could influence hallucination and delirium [22]. In addition, we don’t have granular information about sedation level, coma or use of deep sedation. The use of antipsychotic medications in patients with hallucinations is not surprising given the overlap with delirium. However, as such hallucination often preceded delirium onset, they may be target for preventive pharmacological intervention. We did not specifically focus on the impact of patient sex. However, male prevalence was the same among patients with or without hallucinations. Also, the study patients were not assessed independently for the presence of delirium using a standardised delirium screening tool. However, NLP has been validated to identify such patients similarly to other delirium screening tools [12]. Finally, it is important to consider that this represents the first steps towards developing a new tool for future research. Thus, further validation is needed.

## Conclusion

NLP enables the near real-time, recall bias free, identification of critically ill patients with hallucinations. Using NLP, we found that close to one in 12 patients hallucinated. Moreover, we demonstrated that almost all such patients developed disturbed behaviour, and that patients with hallucinations were much more likely to receive antipsychotic medications. In their aggregate, these observations suggest that hallucinations may be part of the complex phenotypical landscape of critical illness associated neurocognitive dysfunction.

## Supplementary Information

Additional file1 (DOCX 68 KB)

## Data Availability

The data supporting this study will be available for research purposes for 12 months following publication. Access will be granted upon submission of an appropriate research protocol to the corresponding author and subsequent approval by the institutional review boards of both the requesting researcher and the corresponding author.
