# Supplementary material for: Hallucinations and disturbed behaviour in the critically ill: incidence, patient characteristics, associations, trajectory, and outcomes
Source: Crit Care. 2025 Jan 31;29:54. doi: 10.1186/s13054-025-05290-1 (PMC11783843; doi:10.1186/s13054-025-05290-1)
Supplement: Supplementary file 1 — Additional file1 (DOCX 68 KB) [file 13054_2025_5290_MOESM1_ESM.docx]

**Hallucinations and Disturbed Behaviour in the Critically Ill: Incidence, Patient Characteristics, Associations, Trajectory, and Outcomes**

**ONLINE APPENDIX**

| **eTable 1 – Words Used in the Natural Language Processing Analysis** | | | |
| --- | --- | --- | --- |
| **Words suggestive of behavioural disturbance** | | | |
| agitated | agitation | aggression | aggressive |
| combative | confused | confusion | disorientation |
| endangering | disorganized | disorganised | disorientated |
| paranoia | distraction | disturbed | delusion |
| shackled | fluctuating | inattention | incoherent |
| violence | paranoid | restrained |  |
| uncooperative | violent | restraint |  |
| **Negation and Resolution Words** | | | |
| no | not | nil |  |
| resolved | resolving | nill |  |
| ceased | cleared | clearing |  |
| **Words suggestive of hallucinations** | | | |
| hallucinations | halucination |  |  |

| **eTable 2 – Rate of Missing Data** | | | |
| --- | --- | --- | --- |
|  | **Overall**  **(*n* = 7525)** | **Hallucination**  **(*n* = 625)** | **No Hallucination**  **(*n* = 6900)** |
| Age | 0 (0.0) | 0 (0.0) | 0 (0.0) |
| Male gender | 6 (0.1) | 0 (0.0) | 6 (0.1) |
| Body mass index | 6567 (87.3) | 537 (85.9) | 6030 (87.4) |
| APACHE III | 7 (0.1) | 1 (0.2) | 6 (0.1) |
| ANZROD | 47 (0.6) | 3 (0.5) | 44 (0.6) |
| Type of admission | 3 (0.0) | 1 (0.2) | 2 (0.0) |
| Planned admission | 0 (0.0) | 0 (0.0) | 0 (0.0) |
| MET call admission | 1 (0.0) | 1 (0.2) | 0 (0.0) |
| Cardiac arrest | 11 (0.1) | 1 (0.2) | 10 (0.1) |
| Acute renal failure | 54 (0.7) | 4 (0.6) | 50 (0.7) |
| Admission diagnosis | 3 (0.0) | 1 (0.2) | 2 (0.0) |
| ICU source of admission | 0 (0.0) | 0 (0.0) | 0 (0.0) |
| Co-existing disorders |  |  |  |
| Diabetes | 6181 (82.1) | 511 (81.8) | 5670 (82.2) |
| Chronic lung disease | 0 (0.0) | 0 (0.0) | 0 (0.0) |
| Chronic cardiovascular disease | 0 (0.0) | 0 (0.0) | 0 (0.0) |
| Cirrhosis | 0 (0.0) | 0 (0.0) | 0 (0.0) |
| Chronic kidney disease | 0 (0.0) | 0 (0.0) | 0 (0.0) |
| Chronic immune disease | 0 (0.0) | 0 (0.0) | 0 (0.0) |
| Immunosuppression | 0 (0.0) | 0 (0.0) | 0 (0.0) |
| Hepatic failure | 0 (0.0) | 0 (0.0) | 0 (0.0) |
| Lymphoma | 0 (0.0) | 0 (0.0) | 0 (0.0) |
| Metastatic cancer | 0 (0.0) | 0 (0.0) | 0 (0.0) |
| Leukemia | 0 (0.0) | 0 (0.0) | 0 (0.0) |
| Organ support |  |  |  |
| ECMO | 2332 (31.0) | 158 (25.3) | 2174 (31.5) |
| Vasopressor or inotropes | 2330 (31.0) | 158 (25.3) | 2172 (31.5) |
| Invasive ventilation | 1191 (15.8) | 69 (11.0) | 1122 (16.3) |
| Non-invasive ventilation | 2301 (30.6) | 156 (25.0) | 2145 (31.1) |
| Renal replacement therapy | 2194 (29.2) | 139 (22.2) | 2055 (29.8) |
| Laboratory tests |  |  |  |
| pH | 1098 (14.6) | 60 (9.6) | 1038 (15.0) |
| PaO_2_ / FiO_2_ | 1097 (14.6) | 60 (9.6) | 1037 (15.0) |
| PaCO_2_ | 1110 (14.8) | 60 (9.6) | 1050 (15.2) |
| Lactate | 2646 (35.2) | 182 (29.1) | 2464 (35.7) |
| Highest creatinine | 1165 (15.5) | 88 (14.1) | 1077 (15.6) |
| Lowest platelet | 1156 (15.4) | 89 (14.2) | 1067 (15.5) |
| Vital signs |  |  |  |
| Lowest MAP | 1309 (17.4) | 108 (17.3) | 1201 (17.4) |
| Highest RR | 104 (1.4) | 5 (0.8) | 99 (1.4) |
| Highest temperature | 90 (1.2) | 5 (0.8) | 85 (1.2) |
| Urine output | 2489 (33.1) | 169 (27.0) | 2320 (33.6) |
| Clinical outcomes |  |  |  |
| Duration of ventilation | 0 (0.0) | 0 (0.0) | 0 (0.0) |
| ICU length of stay | 9 (0.1) | 1 (0.2) | 8 (0.1) |
| Hospital length of stay | 7 (0.1) | 0 (0.0) | 7 (0.1) |
| ICU mortality | 11 (0.1) | 1 (0.2) | 10 (0.1) |
| Hospital mortality | 4 (0.1) | 0 (0.0) | 4 (0.1) |
| 28-day mortality | 7 (0.1) | 0 (0.0) | 7 (0.1) |

| **eTable 3 – Characteristics of Notes Assessed in the Included Group** | | | | |
| --- | --- | --- | --- | --- |
|  | **Overall**  **(*n* = 7525)** | **Hallucination**  **(*n* = 625)** | **No Hallucination**  **(*n* = 6900)** | ***p* value** |
| Type of hallucination – no. (%) |  |  |  |  |
| Visual | 161 (2.1) | 161 (25.8) | --- | --- |
| Auditory | 41 (0.5) | 41 (6.6) | --- | --- |
| Auditory and visual | 40 (0.5) | 40 (6.4) | --- | --- |
| Days between ICU admission and first episode of hallucination | 2.5 (1.1 - 5.8) | 2.5 (1.1 - 5.8) | --- | --- |
| Patients with recurrent hallucination – no. (%) | 281 (3.7) | 281 (45.0) | --- | --- |
| Median number of episodes per patient with recurrent hallucinations | 3.0 (2.0 - 4.0) | 3.0 (2.0 - 4.0) | --- | --- |
| Hour until resolution of hallucination* | 15.0 (12.0 - 18.0) | 15.0 (12.0 - 18.0) | --- | --- |
| Alternative definition* | 27.0 (24.0 - 37.0) | 27.0 (24.0 - 37.0) | --- | --- |
| Total notes assessed | 11.0 (5.0 - 22.0) | 31.0 (16.0 - 68.0) | 10.0 (5.0 - 19.0) | < 0.001 |
| Number of notes with hallucination | 0.0 (0.0 - 0.0) | 1.0 (1.0 - 3.0) | 0.0 (0.0 - 0.0) | < 0.001 |
| Percentage of notes with hallucination | 0.00 (0.00 - 0.00) | 0.06 (0.03 - 0.10) | 0.0 (0.0 - 0.0) | < 0.001 |
| Data are median (quartile 25^th^ – quartile 75^th^)  Abbreviations: ICU is intensive care unit  * Resolution of hallucination was defined as the first moment when the patient was free of hallucination for at least 12 consecutive hours (or 24 consecutive hours in the alternative definition). | | | | |

| **eTable 4 – Baseline Characteristics of the Included Patients According to Cohort** | | | |
| --- | --- | --- | --- |
|  | **Medication Cohort**  **(*n* = 2904)** | **Rest of the Cohort**  **(*n* = 4621)** | ***p* value** |
| Age, years | 63.8 (50.9 - 74.6) | 63.7 (51.2 - 73.9) | 0.438 |
| Male gender - no. (%) | 1763 (60.8) | 2856 (61.9) | 0.539 |
| Body mass index, kg/m^2^ | 27.8 (24.0 - 32.4) | 27.7 (24.0 - 32.0) | 0.805 |
| APACHE III | 50.0 (37.0 - 67.0) | 46.0 (33.0 - 62.5) | < 0.001 |
| ANZROD | 3.0 (0.8 - 11.1) | 2.1 (0.6 - 9.2) | < 0.001 |
| Type of admission - no. (%) |  |  | 0.001 |
| Medical | 1559 (53.7) | 2295 (49.7) |  |
| Surgical | 1344 (46.3) | 2324 (50.3) |  |
| Planned admission - no. (%) | 826 (28.4) | 1429 (30.9) | 0.023 |
| MET call admission - no. (%) | 545 (18.8) | 755 (16.3) | 0.007 |
| Cardiac arrest - no. (%) | 62 (2.1) | 136 (2.9) | 0.032 |
| Acute renal failure - no. (%) | 73 (2.5) | 143 (3.1) | 0.137 |
| Admission diagnosis - no. (%) |  |  | < 0.001 |
| Cardiovascular | 856 (29.5) | 1636 (35.4) |  |
| Gastrointestinal | 492 (16.9) | 788 (17.1) |  |
| Gynaecological | 8 (0.3) | 6 (0.1) |  |
| Haematological | 29 (1.0) | 39 (0.8) |  |
| Metabolic | 189 (6.5) | 270 (5.8) |  |
| Musculoskeletal/Skin | 80 (2.7) | 79 (1.7) |  |
| Neurological | 282 (9.7) | 360 (7.8) |  |
| Renal/Genitourinary | 147 (5.1) | 214 (4.6) |  |
| Respiratory | 416 (14.3) | 662 (14.3) |  |
| Sepsis | 299 (10.3) | 409 (8.9) |  |
| Trauma | 105 (3.6) | 156 (3.4) |  |
| ICU source of admission - no. (%) |  |  | < 0.001 |
| Emergency department | 747 (25.7) | 1032 (22.3) |  |
| Operating room | 1334 (45.9) | 2311 (50.0) |  |
| Ward | 505 (17.4) | 710 (15.4) |  |
| ICU other hospital | 60 (2.1) | 89 (1.9) |  |
| Other hospital | 250 (8.6) | 474 (10.3) |  |
| ICU same hospital | 6 (0.2) | 0 (0.0) |  |
| Other | 2 (0.1) | 5 (0.1) |  |
| Co-existing disorders - no. (%) |  |  |  |
| Diabetes | 612 (85.7) | 447 (71.0) | < 0.001 |
| Chronic lung disease | 367 (12.6) | 370 (8.0) | < 0.001 |
| Chronic cardiovascular disease | 142 (4.9) | 197 (4.3) | 0.209 |
| Cirrhosis | 227 (7.8) | 333 (7.2) | 0.343 |
| Chronic kidney disease | 317 (10.9) | 362 (7.8) | < 0.001 |
| Chronic immune disease | 34 (1.2) | 158 (3.4) | < 0.001 |
| Immunosuppression | 280 (9.6) | 305 (6.6) | < 0.001 |
| Hepatic failure | 35 (1.2) | 69 (1.5) | 0.312 |
| Lymphoma | 35 (1.2) | 48 (1.0) | 0.499 |
| Metastatic cancer | 141 (4.9) | 189 (4.1) | 0.119 |
| Leukemia | 71 (2.4) | 82 (1.8) | 0.053 |
| Organ support - no. (%) |  |  |  |
| ECMO | 10 (0.3) | 7 (0.3) | 0.813 |
| Vasopressor or inotropes | 1465 (50.9) | 1232 (53.2) | 0.088 |
| Invasive ventilation | 1419 (49.3) | 2487 (72.0) | < 0.001 |
| Non-invasive ventilation | 198 (6.9) | 145 (6.2) | 0.340 |
| Renal replacement therapy | 182 (6.3) | 307 (12.5) | < 0.001 |
| Laboratory tests |  |  |  |
| pH | 7.39 (7.33 - 7.43) | 7.37 (7.31 - 7.42) | < 0.001 |
| PaO_2_ / FiO_2_ | 304 (208 - 400) | 308 (215 - 402) | 0.142 |
| PaCO_2_, mmHg | 40 (35 - 44) | 41 (36 - 46) | < 0.001 |
| Lactate, mmol/L | 2.0 (1.5 - 3.1) | 2.1 (1.5 - 3.3) | 0.085 |
| Highest creatinine, µmol/L | 89 (68 - 137) | 93 (70 - 140) | 0.007 |
| Lowest platelet, x 10^9^/L | 177 (126 - 245) | 175 (125 - 233) | 0.039 |
| Vital signs |  |  |  |
| Lowest MAP, mmHg | 66 (60 - 72) | 65 (58 - 73) | 0.106 |
| Highest RR, breaths/min | 20 (18 - 25) | 18 (15 - 25) | < 0.001 |
| Highest temperature, ºC | 37.2 (36.8 - 37.6) | 37.2 (36.5 - 37.5) | 0.002 |
| Urine output, mL | 1511 (1080 - 2106) | 1503 (1095 - 2176) | 0.502 |
| Hallucination during follow-up – no. (%) | 252 (8.7) | 373 (8.1) | 0.368 |
| Days between ICU admission and first episode of hallucination | 2.7 (1.2 - 6.3) | 2.4 (1.1 - 5.8) | 0.333 |
| Total notes assessed | 11.0 (5.0 - 22.2) | 11.0 (5.0 - 22.0) | 0.212 |
| Number of notes with hallucination | 0.0 (0.0 - 0.0) | 0.0 (0.0 - 0.0) | 0.347 |
| Percentage of notes with hallucination | 0.0 (0.0 - 0.0) | 0.0 (0.0 - 0.0) | 0.362 |
| Clinical outcomes |  |  |  |
| Duration of ventilation, days* | 1.0 (0.5 - 3.6) | 1.1 (0.4 - 4.0) | 0.832 |
| ICU length of stay, days | 1.9 (1.0 - 3.8) | 1.9 (1.0 - 3.8) | 0.811 |
| Hospital length of stay, days | 9.9 (5.5 - 18.0) | 10.0 (5.8 - 19.4) | 0.140 |
| ICU mortality - no. (%) | 160 (5.5) | 325 (7.0) | 0.008 |
| Hospital mortality - no. (%) | 225 (7.7) | 461 (10.0) | 0.001 |
| 28-day mortality - no. (%) | 194 (6.7) | 407 (8.8) | 0.001 |
| Data are median (IQR) or N (%).  * Duration of ventilation reported only in patients who received ventilation.  Abbreviations: APACHE is Acute Physiology and Chronic Health Evaluation; MET is medical emergency team; ICU is intensive care unit; ECMO is extracorporeal membrane oxygenation; MAP is mean arterial pressure; RR is respiratory rate. | | | |

| **eTable 5 – Clinical Outcomes of Included Patients in Medications Cohort** | | | | | | | |
| --- | --- | --- | --- | --- | --- | --- | --- |
|  |  |  |  | **Unadjusted Models** | | **Adjusted Models^a^** | |
|  | **Overall**  **(*n* = 2904)** | **Hallucination**  **(*n* = 252)** | **No Hallucination**  **(*n* = 2652)** | **Effect Estimate**  **(95% CI)** | ***p* value** | **Effect Estimate**  **(95% CI)** | ***p* value** |
| Duration of ventilation, days* | 1.0 (0.5 - 3.6) | 3.9 (1.3 - 8.8) | 0.9 (0.4 - 2.8) | HR, 1.27 (0.18 to 9.06) | 0.814 | HR, 1.35 (0.19 to 9.76) | 0.763 |
| ICU length of stay, days | 1.9 (1.0 - 3.8) | 5.0 (2.7 - 9.9) | 1.8 (0.9 - 3.3) | HR, 1.16 (0.81 to 1.66) | 0.427 | HR, 1.03 (0.72 to 1.48) | 0.854 |
| Hospital length of stay, days | 9.9 (5.5 - 18.0) | 19.7 (10.0 - 32.5) | 9.3 (5.3 - 16.7) | HR, 0.93 (0.64 to 1.36) | 0.727 | HR, 1.05 (0.72 to 1.52) | 0.796 |
| Hospital mortality - no. (%) | 225 (7.7) | 18 (7.1) | 207 (7.8) | HR, 0.92 (0.23 to 3.71) | 0.907 | HR, 0.96 (0.24 to 3.89) | 0.958 |
| 28-day mortality - no. (%) | 194 / 2902 (6.7) | 13 / 252 (5.2) | 181 / 2650 (6.8) | HR, 1.11 (0.27 to 4.47) | 0.883 | HR, 1.02 (0.25 to 4.14) | 0.972 |
| Data are median (quartile 25^th^ – quartile 75^th^) or N (%)  Abbreviations: ICU is intensive care unit; OR is odds ratio; HR is hazard ratio; MD is median difference.  * Duration of ventilation reported only in patients who received ventilation  ^a^ All models adjusted for age, type of admission and ANZROD (after logarithmic transformation) | | | | | | | |

| **eTable 6 – First Medication Administered to Patients During ICU Stay in Medications Cohort** | | | |
| --- | --- | --- | --- |
|  | **Hallucination**  **(*n* = 252)** | **No Hallucination**  **(*n* = 2652)** | ***p* value** |
| Any atypical antipsychotics – no. (%) | 60 (23.8) | 147 (5.5) | < 0.001 |
| Medication – no. (%) |  |  | 0.459 |
| Olanzapine | 8 (13.3) | 30 (20.4) |  |
| Quetiapine | 51 (85.0) | 115 (78.2) |  |
| Risperidone | 1 (1.7) | 2 (1.4) |  |
| Dose, mg | 12.5 (12.5 - 25.0) | 20.0 (12.5 - 25.0) | 0.701 |
| Before first episode of hallucination – no. (%) | 33 (55.0) | --- | --- |
| Days before first episode of hallucination | -2.5 (-5.3 - -0.6) | --- | --- |
| After first episode of hallucination – no. (%) | 27 (45.0) | --- | --- |
| Days after first episode of hallucination | 0.5 (0.3 - 3.9) | --- | --- |
| Other antipsychotics – no. (%) | 30 (11.9) | 51 (1.9) | < 0.001 |
| Haloperidol – no. (%) | 30 (100.0) | 51 (100.0) | --- |
| Dose, mg | 1.5 (1.0 - 2.5) | 1.0 (1.0 - 2.5) | 0.963 |
| Before first episode of hallucination – no. (%) | 21 (70.0) | --- | --- |
| Days before first episode of hallucination | -2.2 (-5.8 - -0.7) | --- | --- |
| After first episode of hallucination – no. (%) | 9 (30.0) | --- | --- |
| Days after first episode of hallucination | 0.9 (0.3 - 1.0) | --- | --- |
| Any antipsychotic – no. (%) | 63 (25.0) | 165 (6.2) | < 0.001 |
| Medication – no. (%) |  |  | 0.408 |
| Haloperidol | 14 (22.2) | 26 (15.8) |  |
| Olanzapine | 7 (11.1) | 26 (15.8) |  |
| Quetiapine | 41 (65.1) | 112 (67.9) |  |
| Risperidone | 1 (1.6) | 1 (0.6) |  |
| Dose, mg | 12.5 (5.0 - 25.0) | 12.5 (5.0 - 25.0) | 0.298 |
| Before first episode of hallucination – no. (%) | 37 (58.7) | --- | --- |
| Days before first episode of hallucination | -2.6 (-5.3 - -1.1) | --- | --- |
| After first episode of hallucination – no. (%) | 26 (41.3) | --- | --- |
| Days after first episode of hallucination | 0.5 (0.3 - 1.7) | --- | --- |
| Ketamine use – no. (%) | 64 (25.4) | 182 (6.9) | < 0.001 |
| Mean dose, mg/h | 9.9 (7.1 - 13.3) | 8.1 (6.0 – 11.0) | 0.022 |
| Before first episode of hallucination – no. (%) | 62 (96.9) | --- | --- |
| Days before first episode of hallucination | -1.0 (-1.8 - -0.5) | --- | --- |
| After first episode of hallucination – no. (%) | 2 (3.1) | --- | --- |
| Days after first episode of hallucination | 12.8 (10.0 - 15.5) | --- | --- |
| Dexmedetomidine use – no. (%) | 77 (30.6) | 152 (5.7) | < 0.001 |
| Mean dose, µg/h | 50.3 (34.4 - 80.1) | 40.8 (24.0 - 62.9) | 0.009 |
| Before first episode of hallucination – no. (%) | 68 (88.3) | --- | --- |
| Days before first episode of hallucination | -3.0 (-6.5 - -1.6) | --- | --- |
| After first episode of hallucination – no. (%) | 9 (11.7) | --- | --- |
| Days after first episode of hallucination | 0.9 (0.4 - 4.7) | --- | --- |
| Data are median (IQR) or N (%).  Abbreviations: ICU is intensive care unit. | | | |

| **eTable 7 – Multivariable Analysis Assessing Baseline Characteristics Independently Associated with the Development of Hallucination** | | |
| --- | --- | --- |
|  | **Odds Ratio**  **(95% CI)** | ***p* value** |
| Age, years | 1.00 (0.99 to 1.01) | 0.585 |
| ANZROD | 0.99 (0.98 to 0.99) | **0.049** |
| Planned admission | 0.91 (0.66 to 1.26) | 0.578 |
| Acute renal failure | 1.70 (1.04 to 2.74) | **0.031** |
| Admission diagnosis |  |  |
| Cardiovascular | 1 (Reference) |  |
| Gastrointestinal | 2.29 (1.59 to 3.29) | **< 0.001** |
| Gynaecological | Not estimated | 0.970 |
| Haematological | 1.40 (0.21 to 5.19) | 0.665 |
| Metabolic | 1.81 (1.00 to 3.23) | 0.048 |
| Musculoskeletal/Skin | 3.59 (1.67 to 7.11) | **< 0.001** |
| Neurological | 1.68 (1.05 to 2.63) | 0.027 |
| Renal/Genitourinary | 1.04 (0.47 to 2.11) | 0.923 |
| Respiratory | 1.16 (0.73 to 1.81) | 0.510 |
| Sepsis | 1.10 (0.65 to 1.80) | 0.723 |
| Trauma | 2.61 (1.37 to 4.76) | 0.002 |
| ICU source of admission - no. (%) |  |  |
| Emergency department | 1 (Reference) |  |
| ICU other hospital | 0.77 (0.38 to 1.53) | 0.474 |
| Operating room | 0.79 (0.53 to 1.18) | 0.244 |
| Other hospital | 0.87 (0.54 to 1.38) | 0.551 |
| Ward | 1.28 (0.86 to 1.88) | 0.222 |
| Other | 0.68 (0.03 to 4.30) | 0.731 |
| Co-existing disorders |  |  |
| Cirrhosis | 1.50 (1.00 to 2.23) | **0.049** |
| Hepatic failure | 0.83 (0.38 to 1.68) | 0.621 |
| Organ support |  |  |
| Vasopressor or inotropes | 1.27 (0.91 to 1.78) | 0.164 |
| Invasive ventilation | 1.59 (1.11 to 2.28) | **0.012** |
| Renal replacement therapy | 2.95 (1.99 to 4.35) | **< 0.001** |
| Laboratory tests |  |  |
| pH | 0.51 (0.14 to 1.90) | 0.318 |
| PaO_2_ / FiO_2_ | 1.00 (1.00 to 1.00) | 0.637 |
| Lactate, mmol/L | 1.05 (1.00 to 1.09) | **0.048** |
| Highest creatinine, µmol/L | 1.00 (1.00 to 1.00) | 0.654 |
| Lowest platelet, x 10^9^/L | 1.00 (1.00 to 1.00) | 0.451 |
| Vital signs |  |  |
| Highest temperature, ºC | 1.19 (1.03 to 1.38) | **0.021** |
| Urine output, mL | 1.00 (1.00 to 1.00) | 0.828 |

| **eTable 8 – Baseline Characteristics of Patients Not Receiving Mechanical Ventilation** | | | | |
| --- | --- | --- | --- | --- |
|  | **Overall**  **(*n* = 2428)** | **Hallucination**  **(*n* = 141)** | **No Hallucination**  **(*n* = 2287)** | ***p* value** |
| Age, years | 64.7 (50.4 - 75.9) | 65.3 (49.5 - 75.3) | 64.7 (50.4 - 76.1) | 0.683 |
| Male gender - no. (%) | 1356 (55.9) | 78 (55.3) | 1278 (55.9) | 0.942 |
| Body mass index, kg/m^2^ | 27.4 (23.7 - 32.1) | 25.9 (24.5 - 27.4) | 27.5 (23.7 - 32.4) | 0.159 |
| APACHE III | 48.0 (35.0 - 63.0) | 55.0 (37.0 - 76.0) | 48.0 (35.0 - 63.0) | 0.001 |
| ANZROD | 3.0 (1.0 - 10.1) | 3.6 (1.3 - 16.7) | 3.0 (1.0 - 9.8) | 0.023 |
| Type of admission - no. (%) |  |  |  | 0.853 |
| Medical | 1652 (68.1) | 95 (67.4) | 1557 (68.1) |  |
| Surgical | 774 (31.9) | 46 (32.6) | 728 (31.9) |  |
| Planned admission - no. (%) | 444 (18.3) | 21 (14.9) | 423 (18.5) | 0.314 |
| MET call admission - no. (%) | 652 (26.9) | 43 (30.5) | 609 (26.6) | 0.328 |
| Cardiac arrest - no. (%) | 10 (0.4) | 2 (1.4) | 8 (0.4) | 0.111 |
| Acute renal failure - no. (%) | 55 (2.3) | 7 (5.0) | 48 (2.1) | 0.039 |
| Admission diagnosis - no. (%) |  |  |  | 0.021 |
| Cardiovascular | 404 (16.7) | 15 (10.6) | 389 (17.0) |  |
| Gastrointestinal | 409 (16.9) | 33 (23.4) | 376 (16.5) |  |
| Gynaecological | 9 (0.4) | 1 (0.7) | 8 (0.4) |  |
| Haematological | 36 (1.5) | 1 (0.7) | 35 (1.5) |  |
| Metabolic | 225 (9.3) | 19 (13.5) | 206 (9.0) |  |
| Musculoskeletal/Skin | 85 (3.5) | 7 (5.0) | 78 (3.4) |  |
| Neurological | 168 (6.9) | 8 (5.7) | 160 (7.0) |  |
| Renal/Genitourinary | 187 (7.7) | 6 (4.3) | 181 (7.9) |  |
| Respiratory | 440 (18.1) | 22 (15.6) | 418 (18.3) |  |
| Sepsis | 373 (15.4) | 19 (13.5) | 354 (15.5) |  |
| Trauma | 90 (3.7) | 10 (7.1) | 80 (3.5) |  |
| ICU source of admission - no. (%) |  |  |  | 0.474 |
| Emergency department | 776 (32.0) | 42 (29.8) | 734 (32.1) |  |
| Operating room | 768 (31.6) | 45 (31.9) | 723 (31.6) |  |
| Ward | 592 (24.4) | 35 (24.8) | 557 (24.4) |  |
| ICU other hospital | 21 (0.9) | 2 (1.4) | 19 (0.8) |  |
| Other hospital | 265 (10.9) | 16 (11.3) | 249 (10.9) |  |
| ICU same hospital | 3 (0.1) | 1 (0.7) | 2 (0.1) |  |
| Other | 3 (0.1) | 0 (0.0) | 3 (0.1) |  |
| Co-existing disorders - no. (%) |  |  |  |  |
| Diabetes | 510 (77.9) | 28 (75.7) | 482 (78.0) | 0.688 |
| Chronic lung disease | 349 (14.4) | 21 (14.9) | 328 (14.3) | 0.806 |
| Chronic cardiovascular disease | 149 (6.1) | 11 (7.8) | 138 (6.0) | 0.367 |
| Cirrhosis | 106 (4.4) | 12 (8.5) | 94 (4.1) | 0.030 |
| Chronic kidney disease | 298 (12.3) | 19 (13.5) | 279 (12.2) | 0.691 |
| Chronic immune disease | 42 (1.7) | 3 (2.1) | 39 (1.7) | 0.733 |
| Immunosuppression | 285 (11.7) | 18 (12.8) | 267 (11.7) | 0.686 |
| Hepatic failure | 26 (1.1) | 5 (3.5) | 21 (0.9) | 0.015 |
| Lymphoma | 40 (1.6) | 4 (2.8) | 36 (1.6) | 0.289 |
| Metastatic cancer | 164 (6.8) | 10 (7.1) | 154 (6.7) | 0.862 |
| Leukemia | 87 (3.6) | 7 (5.0) | 80 (3.5) | 0.347 |
| Organ support - no. (%) |  |  |  |  |
| Vasopressor or inotropes | 356 (14.7) | 26 (18.4) | 330 (14.4) | 0.219 |
| Non-invasive ventilation | 198 (8.2) | 10 (7.1) | 188 (8.2) | 0.752 |
| Renal replacement therapy | 85 (3.5) | 14 (9.9) | 71 (3.1) | < 0.001 |
| Laboratory tests |  |  |  |  |
| pH | 7.40 (7.34 - 7.44) | 7.38 (7.31 - 7.44) | 7.40 (7.34 - 7.44) | 0.119 |
| PaO_2_ / FiO_2_ | 323.8 (240.0 - 410.1) | 310.3 (219.3 - 394.6) | 323.8 (242.9 - 412.2) | 0.149 |
| PaCO_2_, mmHg | 38.0 (33.0 - 43.0) | 39.0 (33.0 - 44.0) | 38.0 (33.0 - 42.0) | 0.422 |
| Lactate, mmol/L | 1.9 (1.3 - 2.9) | 2.2 (1.5 - 3.5) | 1.9 (1.3 - 2.8) | 0.017 |
| Highest creatinine, µmol/L | 90.0 (68.0 - 142.0) | 101.0 (66.5 - 158.5) | 90.0 (68.0 - 140.5) | 0.247 |
| Lowest platelet, x 10^9^/L | 193.0 (140.0 - 258.0) | 173.0 (135.0 - 244.0) | 194.0 (140.2 - 258.0) | 0.041 |
| Vital signs |  |  |  |  |
| Lowest MAP, mmHg | 67.0 (60.0 - 76.0) | 66.5 (60.0 - 76.0) | 67.0 (60.0 - 76.0) | 0.891 |
| Highest RR, breaths/min | 23.0 (20.0 - 28.0) | 25.0 (20.0 - 28.0) | 22.0 (20.0 - 28.0) | 0.040 |
| Highest temperature, ºC | 37.0 (36.5 - 37.5) | 37.1 (36.6 - 37.6) | 37.0 (36.5 - 37.5) | 0.101 |
| Urine output, mL | 1500 (1040 - 2205) | 1435 (1016 - 2015) | 1500 (1040 - 2225) | 0.160 |
| Days between ICU admission and first episode of hallucination | 1.1 (0.6 - 1.8) | 1.1 (0.6 - 1.8) | --- | --- |
| Total notes assessed | 8.0 (4.0 - 15.0) | 15.0 (10.0 - 26.0) | 8.0 (4.0 - 14.0) | < 0.001 |
| Number of notes with hallucination | 0.0 (0.0 - 0.0) | 1.0 (1.0 - 2.0) | --- | --- |
| Percentage of notes with hallucination | 0.0 (0.0 - 0.0) | 0.1 (0.1 - 0.1) | --- | --- |
| Data are median (IQR) or N (%).  Abbreviations: APACHE is Acute Physiology and Chronic Health Evaluation; MET is medical emergency team; ICU is intensive care unit; ECMO is extracorporeal membrane oxygenation; MAP is mean arterial pressure; RR is respiratory rate. | | | | |

| **eTable 9 – Clinical Outcomes of Patients Not Receiving Mechanical Ventilation** | | | | | | | |
| --- | --- | --- | --- | --- | --- | --- | --- |
|  |  |  |  | **Unadjusted Models** | | **Adjusted Models^a^** | |
|  | **Overall**  **(*n* = 2428)** | **Hallucination**  **(*n* = 141)** | **No Hallucination**  **(*n* = 2287)** | **Effect Estimate**  **(95% CI)** | ***p* value** | **Effect Estimate**  **(95% CI)** | ***p* value** |
| ICU length of stay, days | 1.6 (0.8 - 2.7) | 2.8 (1.7 - 4.0) | 1.5 (0.8 - 2.6) | HR, 1.06 (0.94 to 1.59) | 0.767 | HR, 1.03 (0.69 to 1.54) | 0.885 |
| Hospital length of stay, days | 8.5 (4.7 - 16.2) | 11.0 (6.6 - 21.9) | 8.3 (4.6 - 15.9) | HR, 0.80 (0.53 to 1.22) | 0.313 | HR, 0.94 (0.62 to 1.43) | 0.777 |
| Hospital mortality - no. (%) | 128 / 2426 (5.3) | 8 / 141 (5.7) | 120 / 2285 (5.3) | HR, 1.61 (0.40 to 6.51) | 0.505 | HR, 1.24 (0.31 to 5.06) | 0.760 |
| 28-day mortality - no. (%) | 106 / 2426 (4.4) | 6 / 141 (4.3) | 100 / 2285 (4.4) | HR, 2.01 (0.50 to 8.16) | 0.327 | HR, 1.72 (0.42 to 6.99) | 0.445 |
| Data are median (quartile 25^th^ – quartile 75^th^) or N (%)  Abbreviations: ICU is intensive care unit; HR is hazard ratio.  * Duration of ventilation reported only in patients who received ventilation  ^a^ All models adjusted for age, type of admission and ANZROD (after logarithmic transformation) | | | | | | | |

**eTable 10 – Typical nursing notes used by NLP assessment to diagnose the presence of hallucinations**

**Note 1**

Day  ICU Day 5 post ed admission  Shift Issues Patient has complained of pain in L) wrist, small radial fracture was identified. L) radial ART line was removed and Backslab plaster cast was administered.  CNS Patient is alert, orientated to person and place, disorientated to time. Patient has displayed some short term memory lapses. RMO informed. Patient has moderate weakness in all four limbs, and slightly weaker in L) upper limb because of fracture and pain. PEARL size 3mm. Pain in L) wrist was relieved with 5mg oral Oxynorm. AWS shows low scores and relieved with supportive care. PEARL size 3mm. Patient says he was experiencing some **visual hallucinations**. like spiders and slugs in the bed. RMO informed, and reassurance provided and patient was relieved.  CVS

**Note 2**

Day 33 in ICU for mx post paracetamol OD and fulminant hepatic failure. CNS Pt eye opening spontaneously or to speech. More alert and interactive this shift. Obeying commands - able to poke out tongue, squeeze with both hands, moving r) leg  Severely weak. PEARL size 5. Denies pain but indicating experiencing anxiety and **auditory hallucinations**. Additional quetiapine doses given with minimal effect. Pt intermittently agitated, attempting to sit forwards, swinging leg over side of the bed. Requiring reassurance +++.

**Note 3**

Pt complaining to nursing staff overnight of **visual and auditory hallucinations**. At time becoming distressed with this, and frustrated with irritation from tracheostomy. RR up to ~40. Then settles back to ~30 (has been for many days.) I note pt has prolonged ICU stay and is on mirtazapine, amitriptylline and quetiapine (ceased 31/10.) ?May require PRN medication for delirium/ agitation.

**Note 4**

Day Day 2 post Ivor Lewis oesphagectomy and wedge resection of RUL lung lesion.  Shift Issues Nil issues for the duration of the shift. CNS pt is drowsy. easily rousable to voice. oriented to time/place/person. Says he has been experiencing **visual hallucinations** on and off throughout the shift however has remained oriented to time/place/person throughout them. PEARL. c/o minimal pain at rest 2-3/10. c/o 6/10 pain on movement.  IV PCA fentanyl in situ - minimal use by patient despite encouragement prior to moving.  IV cont ketamine @ 8ml/hr.

**Note 5**

Day 3 Night shift of day 3 in ICU for behavior management secondary to GHB withdrawal Shift Issues Agitation Deliriium CNS Delirium fluctuating. Initially orientated, appropriate and had insight to cause of hospitalisation. Between 2100-2200 I was able to release wrist restraints After 2200 Damien has become increasingly delirious At times aggressive and verbally abusive Impulsive, trying to climb out of bed Pulling at IDC Damien says he is having **visual hallucinations**. At times thinking there are animals around him, 'things climbing on celling'. At other times he believes I am his brother and that he is currently involved in a car jacking. Mostly incomprehensible however his speech can be understood occasionally. Damien will usually settle down with frequent reorientation and explanation of why he is restrained. A total of 60mg of oral diazapam administer with minimal effect State dose of 10mg of olanzapine administered - effect appears minimal PEARL 3-4mm Moving all four limbs No sleep overnight despite decreased stimulus and constant reassurance.

**Note 6**

ICU CWR (Hart et al) # mastoiditis/cerebritis R mastoidectomy # drop in GCS and tremors on ward triggering MET call multiple differential diagnosis: ?cefepime related ?hypertensive ?inflam/infective LP bland multiple CTB and MRB - unchanged venous sinus thrombosis, small empyema, temporal lobe cerebritits (radiological changes present prior to drop in GCS) currently on vancomycin and meropenem - last vanc level 40 this am # untreated Hep C last viral load >2 million # ?coffee ground vomiting on pantoprazole 80mg BD IV OE: alert, interactive and agitated this morning **visual hallucination** present - 'everyone double headed' extubated at around 10am lungs clear SaO2 100% on RA post extubation Plan: extubated liaise with LTU re: immunosuppressants -

**Note 7**

Day 4 ICU stay with mastoiditis and venous sinus thrombosis BG OLTx 2013/HepC/ETHO cirrhosis  Shift Issues patient reports ongoing **visual hallucinations**  CNS RTVS, RTVC, able to move all limbs equally and spontaneously. Orientated to person, place and time. PEARL 3-4mm. Denies pain, lower back discomfort present, relieved with regular repositioning. NASI A1. VIsual hallucinations present, more obvious when approaching patient from RHS. Seeing blood on hands, big shadow in room and other odd things throughout shift - reorientated and reasurance ++ given, pt quite emotional.

**Note 8**

Progress NGT out this morning (prior to review) Upgraded to FWD/THIN remains on tds sustagen    Clinical patient reports **visual hallucinations** appetite good Biochemistry U&E 143/3.6L Rx/11.2/126; Alb 25L; Hb 95L; WCC 3.5 Anthropometry Nil new, underweight ++ recent weight loss SGA C Dietary (Percentage prescribed nutrition received since

last r/v)

**Note 12**

Day 1 in ICU Shift Issues Sodium rising too quickly Reviewed by Endo (home team) and Psych CNS GCS 14-15. Initially confused to day of week and DOB. Now orientated to time, place and person. Obeying commands. Moving all four limbs, equal limb strength. Ambulant to toilet with supervision. Denies pain. PEARL 3 mm sluggish response to light - ICU aware. Patient reports to Endocrinology team of having **auditory hallucinations** yesterday about aliens. Nil reports of hallucinations this morning but advised staff she has had previous suicidal ideations.
